# Intercropped relationship change the developmental pattern of apple and white clover

**DOI:** 10.1080/21655979.2019.1621137

**Published:** 2019-06-11

**Authors:** Jianfeng Yang, Tairan Zhang, Yuanji Wang, Rongqin Zhang, Huike Li

**Affiliations:** a College of Natural Resources and environment, Northwest A&F University, Yangling, Shaanxi, China; b Key Laboratory of Plant Nutrition and the Agro-Environment in Northwest China, Ministry of Agriculture, Yangling, China

**Keywords:** Apple, intercropping, biomass, root morphology

## Abstract

Intercropping can introduce greater plant diversity and functional complementarity in an arable crop system but inter- and intracompetition can between intercropped crops. The rhizo-box was established of apple-white clover intercropped system to examine the competitive relationship between intercropped crops on the Loess Plateau. The results showed that the competitive relationship between intercropped crops was dynamic and changed with the crop competitiveness. Crop competitiveness was characterized by root development, although intercropping inhibited the development and nutrient accumulation aboveground of apple trunks and branches, intercropped apples still maintained a larger root system than under monoculture and the root morphology of intercropped apples changed significantly. White clover had lower competitiveness than apple at the beginning of the year, which was reflected in the inhibited development in May. However in July and October, intercropped white clover had more biomass and nutrient accumulation than under monoculture.

## Graphical Abstract

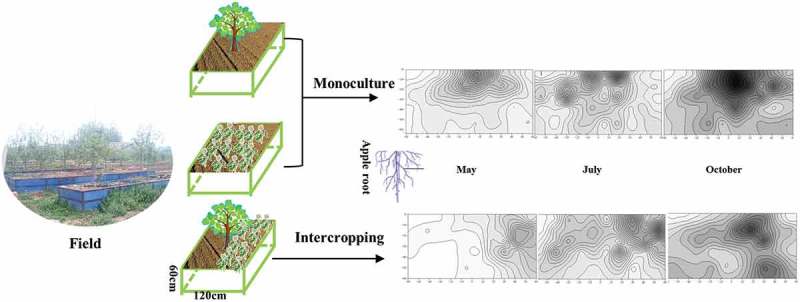

## Introduction

Intercropping is the simultaneous cultivation of two or more crop species in the same field [1] and can introduce greater plant diversity and functional complementarity in arable crop systems [2]. Because the Loess Plateau has been suffered many serious environmental problems such as soil erosion [3], substantial soil organic matter losses, nutrient imbalances, and water deficits, agricultural management, especially intercropping can have strong sustained beneficial effects on soil chemical properties, and microbial communities [4]. Consequently, these problems can be effectively alleviated [5–7], which makes intercropping one of the most important agricultural management measures on the Loess Plateau. Moreover, because one of the largest apple production areas in China is located in this region, intercropping using some crops as living mulch such as white clover (*Trifolium repens* L.) and crown vetch (*Coronilla varia* L.), has been used for decades in this region’s apple orchards to solve those environmental problems [8,9].

Although many intercropping systems have numerous benefits for soil and crops [10,11]，some scholars found intercropping can result in competition between the intercropped crops. This competition has been found in many forms of intercropping, such as relay intercropping [12] and tree-crop systems [13]. Competition caused by intercropping can retard crop development and nutrient absorption [14] and may decrease the yield of the whole systems sometimes [15], results in significant different effects of intercropping. The situation has also occurred in the Loess Plateau apple orchard tree-grass systems but has not been widely reported.

Many factors, including component combinations, total planting densities, and management regimes, can affect this type of competition [16], but the above-mentioned studies of competition in intercropping were mostly static. Zhang et al. found that older tree-ages jujube tree would exert more negative influences than younger tree on aboveground and belowground biomass of wheat in a jujube-wheat system which proved that competition caused by intercropping was probably a dynamic process, which could explain why the negative effects caused by intercropping mostly occurred in young tree crops or initial growth periods of annual crops [13,14]. Most studies have concentrated on one main crop in intercropping systems rather than the entire systems, especially in tree-grass systems such as the Loess Plateau apple orchards, when grass only takes on the role of improving the ecological environment without actual economic output into the system [17].

Additionally, there were some contradictory situations observation that accompanied by these disadvantages of intercropping such as an increase of in crop photosynthesis accompanied by a decrease in leaf traits (nitrogen concentration and chlorophyll). In addition, to explain these contrasting results, researchers reported that intercropping changed the morphological development of maize ear leaves and speculated on changes in the morphological development of maize roots. A change in root morphological development (root distribution) in intercropping systems has been proven by many scholars working on jujube-wheat [13], wheat-maize [18], faba bean-maize [19] and white clover-apple systems. Combined with changes in the root morphological development, changes in the morphological development of other crop organs prove that in addition to a change in the resource acquisition strategy, intercropping probably also changes the crop development strategy. Only some researchers have linked these two phenomena together with respect to transformed of crop roots or other organs and the inevitable effect of slowing crop development and nutrient accumulation.

Interspecific relationship research was the keynote of agroforestry research. Currently, research about the underground interspecific relationship was still weak. Until now, in the apple-grass intercropping system, the mechanism about how apple trees and pasture components affect their mutual nutrition competition or synergism by the root distribution with morphological change and physiological change was still not clear. Based on the above knowledge gaps, we established a rhizo-box of apple, white clover and apple (*Malus ×domestica* cv.)-white clover (*Trifolium repens*) intercropped systems on the Loess Plateau, using 2-year-old apple trees, and measured the biomass and nutrient (nitrogen, phosphorus and potassium) accumulation of the two species in the intercropped systems and the root distribution of the apple trees three times a year (May, July and October). We attempted to verify the following hypotheses: (i) intercropping of 2-year-old apple and white clover would significantly decrease the nutrient uptake and development of apple; (ii) this decrease will be enhanced over time (with the development of white clover); and (iii) one possible reason for the decrease in apple nutrient input and development is a change in the apple development pattern change, and this change is mainly reflected in the change of root morphology.

## Materials and methods

### The study site and treatments

The experiment was conducted at the Northwest A&F University Apple Experimental Station, Baishui County (35°21′N, 109°30′W; elevation 850 m), Weibei Loess Plateau, Shaanxi Province, China. The local climate belongs to a temperate continental climate and conducive to producing high-quality apples due to ample sunshine. The local average annual rainfall amounts to 577.8 mm, with over 80% falling between June and September, and the site’s average annual temperature is 11.4 ℃, with larger yearly fluctuations. Rhizo-boxes have been established at local experimental stations using local soil that had a water-soluble organic carbon, available phosphorus, available potassium, nitrate nitrogen and ammonium nitrogen contents of 0.041, 14.88, 160.80, 22.41 and 3.98 mg/kg, respectively; the soil was classified as a Calcic Cambosol according to the Chinese Soil Taxonomy (CST) [20].

The rhizo-box was built by a PVC (polyvinyl chloride) plate with a length of 120 cm, width of 120 cm and height of 60 cm. Three treatments were established: monoculture apple (*Malus*×*domestica* cv.), monoculture white clover (*Trifolium repens*) and an apple-white clover intercropped system. The apple used in these treatments was a 2-year-old ‘Fuji’ cultivar, grafted on a self-rooted M26 rootstock. To reduce the systematic error caused by the growth of the nursery stock, seedlings that had a similar size and root development were chosen, with an average seedling height of 1.5 m and an average diameter of 1.5 cm. White clover covered half of the surface of every rhizo-box under monoculture and in the white clover and apple-white clover intercropped system, and the sowing density of white clover (*Trifolium repens*) in the covered area was 5 g/m^2^. Apples were planted in the center of the rhizo-box in the monoculture apple and intercropped system treatments. Thirty rhizo-boxes were established in March 2016, including the planting of apple and the sowing of white clover. Each treatment was established in 10 rhizo-boxed. Nine rhizo-box, i.e., including three treatments were randomly selected for determination in May, July and October.

### Sampling

For apple sampling, apple trees were felled to collect all of the above-ground organs, including leaves, shoots, branches and trunks. Then, the fresh weight of each of apple organs was measured at the experiment station. After 30 min at 105 ℃ in an oven, sufficient samples of these organs were dried in the same oven at 80 ℃ to constant weight. The mass of different organs before and after drying was recorded to calculate the water content and then calculate the biomass of different apple organs using fresh weight data. For white clover sampling, three 20 × 20 cm quadrats were chosen by the three-point method, and all white clover plants in the quadrat were mowed in order to measure the fresh weight of leaves and branches. Then, similar to apple sampling, sufficient samples of white clover were subjected to the same process to obtain biomass data and enough homogenized powder for nutrient analysis.

The root sampling method was previously described [21]. After the above-ground parts of the crops were sampling, the PVC plate on the one side of the rhizo-box was removed. Except for the rhizo-box under monoculture white clover treatment, the soil in a rhizo-box was divided into 50 parts and these parts were removed from the rhizo-box one by one. After removing one part of the soil from the rhizo-box, a clean wire brush was used to remove loose soil particles. After removing soil particles in each part, all root samples were placed in a sterile plastic bag, labeled with the treatment and spatial location, sealed and then transported to the laboratory on ice [22].

In the rhizo-box under monoculture white clover treatment, after selecting three quadrats were selected and mowing the white clover in quadrats was moved, the 0–40 cm soil (the main distribution region of white clover roots) in tree quadrats was removed from the rhizo-box. Similar to the sampling of apple roots.

### Determination

The root samples were sprayed with distilled water to remove surface dust in the laboratory and were dried in an oven using the same process as other organs. After drying to constant weight, roots samples were weighed to record the biomass of roots in different treatments and spatial locations. Before these processes, the root samples were spread out for scanning using a WinRhizo (2004b, Regent Instruments Inc., USA) system to distinguish diameter. The fine roots (diameter <1 mm) and thick roots (diameter >1 mm) were regarded as two samples, and then were dried and weighed [23,24].

All plant samples after these processes were ground by a high-throughput tissue grinder (MM400, Retsch Co., Germany), then passed through a 1 mm mesh sieve, homogenized, subdivided, and finally stored in plastic bags at 4℃ until needed for nutrient analysis similar other organs [25]. The sample elements parameters were also analyzed in each plant subsample: total plant nitrogen and phosphorus concentration was analyzed with a flow-injection autoanalyzer (AA3, Seal Co., Germany), total plant potassium concentration was analyzed using flame photometry (M410, SHERWOOD Co., Britain) [26].

### Data analysis

Root biomass density (RBD) = TB/Vs

Here, TB is the dry weight (biomass) of roots in soil and Vs is the volume of this soil [27].

Nutrient accumulation (g/kg) = nutrient content × biomass (dry mass)

**Figure 1.**
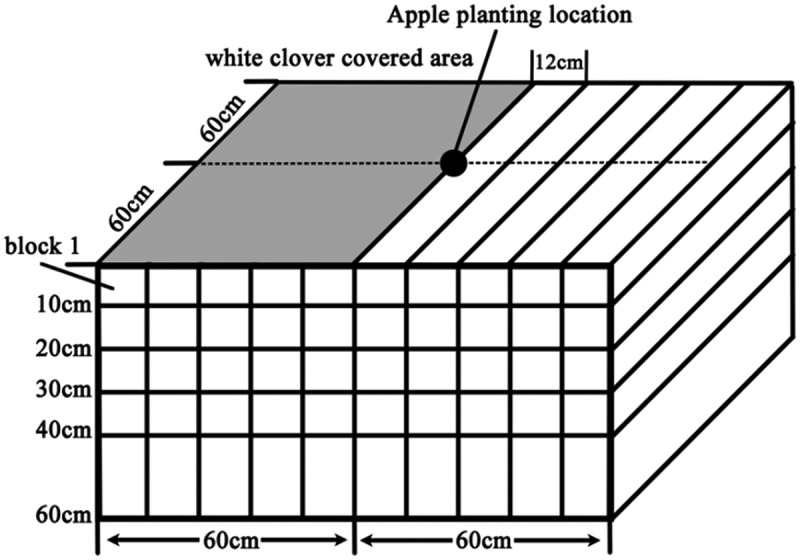
Schematic diagram of apple root sampling.

The results were statistically analyzed using a *P*< 0.05 level of significance. The data were analyzed by one-way ANOVA using the SPSS 20.0 software package (2010, V. 19.0, SPSS Inc., Cary, NC, USA), and the least significant difference (LSD) test was used to identify significant differences. Isoline maps were created using the Surfer 11 software package based on RBD data from the apple and Kriging methods. In the isoline map, the RBD value of a soil block’s was defined as the value in the center of this soil block physical location (e.g., for the soil block 1 shown in Figure 1, the coordinate of its RBD value on the isoline map was 6,-5).

## Results

### Crop development

The development of apple was significantly inhibited by intercropping with white clover (Table 1). The inhibition of apple leaves mainly occurred in July, which could reflect a major growth period of apple, i.e., the growing of autumn shoots, on the Loess Plateau. There were no significant differences between monoculture apple leaves and intercropped apple leaves in October because October mainly reflects the apple deciduous period when apple development slows down in preparation for the local winter. Furthermore, in May, the period of growth for Spring shoots, intercropping significantly promoted the development of apple leaves. Intercropping with white clover inhibited apple branch and trunk development, but the development of apple roots showed different patterns: in July and October, intercropping with white clover did not decrease apple root biomass, and in May and October, biomass increased.

**Table 1.** The biomass of apple and white clover in three seasons.

	Apple biomass g/tree					
	May		July		October	
Organs	Monoculture	Intercropping	Monoculture	Intercropping	Monoculture	Intercropping
Leaves	67.05 ± 6.72	59.72 ± 0.66	217.99 ± 49.46	149.68 ± 21.78*	167.90 ± 0.28	86.55 ± 8.57**
Shoot	10.45 ± 0.23	10.50 ± 0.20	55.27 ± 5.07	61.85 ± 2.40	49.90 ± 19.14	39.06 ± 3.71
Branch	150.36 ± 13.30	178.99 ± 55.60	595.64 ± 31.52	341.28 ± 33.75**	464.57 ± 102.22	306.73 ± 7.88*
Trunk	152.31 ± 13.29	125.00 ± 4.34*	562.66 ± 34.18	430.29 ± 24.50**	499.47 ± 92.85	300.50 ± 33.45**
Roots	141.37 ± 21.16	91.11 ± 12.73*	173.59 ± 1.13	205.29 ± 1.72 **	408.53 ± 55.16	316.47 ± 8.68*
Total	520.39 ± 76.14	456.26 ± 64.23	1626.75 ± 94.23	1204.62 ± 78.08**	1579 ± 322.40	1050.70 ± 55.94
White Clover biomass g/m^2^						
Leaves	747.26 ± 16.64	671.44 ± 35.81*	294.42 ± 23.26	492.96 ± 17.52**	143.47 ± 6.47	184.52 ± 11.97**
Branch	847.92 ± 41.07	842.03 ± 68.39	338.07 ± 0.94	572.18 ± 10.92**	186.14 ± 54.98	293.04 ± 62.68
Thick roots	61.22 ± 22.71	33.33 ± 3.71*	7.76 ± 0.65	10.46 ± 3.51	9.85 ± 1.91	9.22 ± 3.86
Fine Roots	81.28 ± 28.62	26.10 ± 3.12**	9.43 ± 1.39	13.84 ± 4.47	26.82 ± 4.78	17.60 ± 0.82*
Total	1736.01 ± 33.34	1540.55 ± 76.25**	649.68 ± 25.36	1089.44 ± 20.79**	366.28 ± 55.25	504.39 ± 49.25*

* indicates that correlations are significant (p < 0.05), ** indicates that correlations are significant (p < 0.01)

Intercropping with apple significantly inhibited white clover development (Table 1), but only in May, which was mainly embodied in leaves and the total biomass decrease of white clover. However, in July and October, there was no inhibition, and intercropping with apple even promoted white clover branch and leaf development and increased the total biomass of white clover. In October, only fine root development of white clover was inhibited in this intercropped system.

### Crop nutrient accumulation

The nutrient accumulation of apple was increased when white clover was declined, and intercropping changed the nutrient accumulation of the two crops (Table 2 and Table 3). In May, intercropping increased phosphorus accumulation in apple leaves and potassium accumulation in apple shoots but decreased nutrient accumulation in apple trunk and roots, and intercropping generally decreased almost all three nutrient accumulation in white clover. In July, intercropping significantly decreased apple above-ground nutrient accumulation, but this inhibition on nutrient accumulation did not occur in the belowground part of apple when intercropping promoted nutrient accumulation in white clover, although the promotion of white clover root nutrient accumulation did not reach a significant level. These effects, the promotion of white clover nutrient accumulation and the inhibition of apple nutrient accumulation, weakened in October, and the main observations were only total nitrogen accumulation and leaf potassium accumulation of intercropped white clover were significantly higher than those of white clover under monoculture management. In fact, potassium accumulation of whiter clover roots was lower than that under monoculture when intercropping no longer inhibited apple leaves and shoot nutrient accumulation.

**Table 2.** Nutrient (total nitrogen, phosphorus and potassium) accumulation of apple.

Organs	Management	N g/kg	P g/kg	K g/kg
May				
Leaves	Monoculture	0.502 ± 0.015	0.044 ± 0.001	0.259 ± 0.008
	Intercropping	0.501 ± 0.007	0.047 ± 0.001*	0.255 ± 0.003
Shoots	Monoculture	0.307 ± 0.007	0.031 ± 0.001	0.153 ± 0.003
	Intercropping	0.297 ± 0.006	0.031 ± 0.001	0.172 ± 0.003**
Branch	Monoculture	1.156 ± 0.102	0.134 ± 0.012	0.839 ± 0.074
	Intercropping	1.286 ± 0.506	0.197 ± 0.077	1.108 ± 0.436
Trunk	Monoculture	0.794 ± 0.069	0.122 ± 0.011	0.509 ± 0.044
	Intercropping	0.639 ± 0.022*	0.089 ± 0.003**	0.499 ± 0.017
Roots	Monoculture	2.145 ± 0.321	0.289 ± 0.043	0.177 ± 0.027
	Intercropping	1.088 ± 0.152**	0.189 ± 0.026*	0.104 ± 0.014*
Total	Monoculture	4.904 ± 0.128	0.620 ± 0.019	1.937 ± 0.103
	Intercropping	3.811 ± 0.454	0.552 ± 0.069	2.137 ± 0.435
July				
Leaves	Monoculture	0.803 ± 0.064	0.039 ± 0.003	0.389 ± 0.031
	Intercropping	0.408 ± 0.009**	0.028 ± 0.001**	0.295 ± 0.007**
Shoots	Monoculture	1.153 ± 0.106	0.087 ± 0.008	0.482 ± 0.044
	Intercropping	1.644 ± 0.064**	0.108 ± 0.004*	0.552 ± 0.021
Branch	Monoculture	5.424 ± 0.287	1.058 ± 0.056	2.503 ± 0.132
	Intercropping	2.686 ± 0.266**	0.619 ± 0.061**	1.789 ± 0.177**
Trunk	Monoculture	3.015 ± 0.183	0.676 ± 0.041	1.679 ± 0.102
	Intercropping	2.103 ± 0.120**	0.543 ± 0.031*	1.263 ± 0.072**
Roots	Monoculture	2.968 ± 0.019	0.446 ± 0.003	1.451 ± 0.009
	Intercropping	2.958 ± 0.025	0.467 ± 0.00	1.381 ± 0.012
Total	Monoculture	13.363 ± 0.492	2.305 ± 0.099	6.505 ± 0.238
	Intercropping	9.800 ± 0.415**	1.765 ± 0.092**	5.280 ± 0.252**
October				
Leaves	Monoculture	0.229 ± 0.046	0.026 ± 0.005	0.102 ± .0.020
	Intercropping	0.226 ± 0.011	0.026 ± 0.001	0.110 ± 0.006
Shoots	Monoculture	0.830 ± 0.200	0.116 ± 0.028	0.270 ± 0.065
	Intercropping	0.504 ± 0.048	0.071 ± 0.007	0.229 ± 0.022
Branch	Monoculture	5.443 ± 0.596	0.763 ± 0.084	2.538 ± 0.278
	Intercropping	3.487 ± 0.090**	0.541 ± 0.014*	1.552 ± 0.040**
Trunk	Monoculture	5.526 ± 0.649	0.788 ± 0.093	1.908 ± 0.224
	Intercropping	2.762 ± 0.415**	0.358 ± 0.054**	0.953 ± 0.143**
Roots	Monoculture	5.765 ± 0.778	1.068 ± 0.144	1.771 ± 0.239
	Intercropping	4.279 ± 0.117*	0.777 ± 0.021*	1.456 ± 0.040
Total	Monoculture	17.793 ± 1.094	2.760 ± 0.189	6.588 ± 0.281
	Intercropping	11.258 ± 0.652**	1.773 ± 0.093**	4.299 ± 0.237**

* indicates that correlations are significant (p < 0.05), ** indicates that correlations are significant (p < 0.01)

**Table 3.** Nutrient (total nitrogen, phosphorus and potassium) accumulation of white clover.

Organs	Management	N g/m^2^	P g/m^2^	K g/m^2^
May				
Leaves	Monoculture	47.446 ± 1.057	3.883 ± 0.086	37.897 ± 0.844
	Intercropping	28.192 ± 1.504**	2.112 ± 0.113**	18.152 ± 0.968**
Branch	Monoculture	30.556 ± 1.047	3.447 ± 0.118	31.255 ± 1.071
	Intercropping	26.445 ± 1.520*	2.570 ± 0.148**	31.989 ± 1.839
Roots	Monoculture	5.161 ± 1.310	0.416 ± 0.106	2.962 ± 0.752
	Intercropping	1.353 ± 0.024**	0.125 ± 0.002**	0.812 ± 0.014**
Total	Monoculture	83.163 ± 1.334	7.746 ± 0.077	72.114 ± 0.561
	Intercropping	55.990 ± 3.016**	4.807 ± 0.260**	50.953 ± 2.802**
July				
Leaves	Monoculture	13.801 ± 1.090	1.161 ± 0.092	12.976 ± 1.025
	Intercropping	24.577 ± 0.876**	2.069 ± 0.074**	21.709 ± 0.772**
Branch	Monoculture	10.153 ± 0.028	1.141 ± 0.003	9.904 ± 0.028
	Intercropping	20.280 ± 0.387**	2.038 ± 0.039**	20.426 ± 0.390**
Roots	Monoculture	0.381 ± 0.044	0.040 ± 0.005	0.186 ± 0.021
	Intercropping	0.629 ± 0.207	0.085 ± 0.028	0.375 ± 0.123
Total	Monoculture	24.335 ± 1.140	2.342 ± 0.097	23.066 ± 1.057
	Intercropping	45.486 ± 1.061**	4.192 ± 0.086**	42.510 ± 1.042**
October				
Leaves	Monoculture	7.154 ± 0.323	0.554 ± 0.025	3.568 ± 0.161
	Intercropping	7.373 ± 0.478	0.568 ± 0.037	4.262 ± 0.277**
Branch	Monoculture	5.453 ± 1.611	0.746 ± 0.220	3.401 ± 1.005
	Intercropping	8.844 ± 1.892	1.027 ± 0.220	4.589 ± 0.982
Roots	Monoculture	0.986 ± 0.180	0.130 ± 0.024	0.615 ± 0.112
	Intercropping	0.700 ± 0.084	0.096 ± 0.011	0.407 ± 0.049*
Total	Monoculture	13.593 ± 1.471	1.430 ± 0.219	7.584 ± 0.957
	Intercropping	16.917 ± 1.402*	1.691 ± 0.177	9.257 ± 0.700

* indicates that correlations are significant (p < 0.05), ** indicates that correlations are significant (p < 0.01)

### Morphological development of apple roots

**Figure 2.**
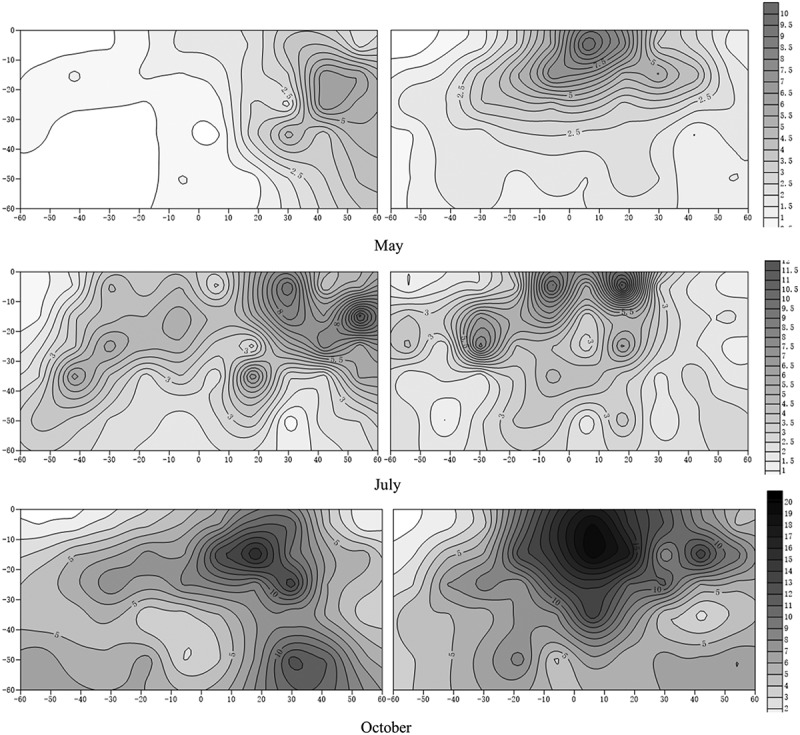
Isoline map of apple root distribution. 1. Abscissa from −50 to 0 (under intercropping) is the area covered by white clover; 2. The absolute value of the ordinate represents the depth of the soil layer; 3. The coordinates (0, 0) are the planting positions of the apple tree

Intercropping also changed the morphological development of apple roots, as shown in Figure 2. The root system of apple under monoculture was obviously radial in three seasons, when intercropped apple had obviously different root architecture. In May, the roots of the intercropped apple mainly developed in the region far away from white clover roots. In July, in addition to root development been maintained in the region far away from white clover roots, intercropped apples produced some roots in the intensively distributed regions of white clover roots. In October, except for these two characteristics mentioned above, apple developed more roots in deeper soil, especially in 50–60 cm soil. In summary, intercropping significantly changed the spatial development strategy of apple roots in the system.

## Discussion

The first hypothesis that intercropping significantly decreases nutrient input and the development of apple has been proven in Table 1, similar to previous studies [14,15]. Because of the inhibition of crop development mainly occurred in the early stage of the crops, especially when the nutrition acquisition ability of the crops was low. We speculate that the competition relationship under the intercropping system will change dynamically with the development of crop competitiveness. Although intercropping significantly inhibited apple development, apple gave prioritized root development, at the expense of the growth of trunks and branches. Therefore, the competitiveness of crops, especially in arid and semiarid regions, is likely related to the development of crop roots. In conclusion, because the development cycle of white clover was much shorter than that of apple, in the early stage of intercropping white clover was more competitive, which led to the inhibition of apple development. However, when apple growth is inhibited, apples will preferentially develop larger root systems and will then have deeper and wider root systems after maturity, based on a previous study [9]. In other words, although the apple in an intercropped system has fewer available resources for apple because of other were another species in the system, apple still maintains its root and shoot development as far as possible. In addition, because the intercropping system in the present study was established in a region where the competition belowground was the main form of intercropped plant [23–25, 28-30] so we guess in a region with adequate soil nutrients, competition for sunlight will become the main form of the competition. In the present study, apple also prioritized the development of shoots according to Table 1, and morphological changes in the photosynthetic rate of maize leaves were found in another study [14].

For white clover, all changes in biomass and nutrient accumulation caused by intercropping indicated the same phenomena, i.e., the postponement of development. Because white clover was annual crop when apple was perennial, the nutrition acquisition ability of white clover was lower than apple at the beginning of the year, which led to the inhibition of white clover development and nutrient accumulation affected by intercropping. However, intercropped white clover maintained more biomass and nutrients in the fall, which showed development postponement of white clover. Similar conditions occurred in a relay cropping system with wheat and maize called compensatory growth [26, 31]. However, because we only measured the biomass of the white clover population, it was not possible to determine whether this phenomenon is in fact compensatory growth. We can only conclude that the development strategies of apple and white clover under intercropping were different from those under monoculture, and in 1 year, apple mainly showed a change in the developmental organs while white clover showed a change in the developmental time.

Intercropping is usually mainly based on two species differences: different resource strategies and different growing periods [27, 32]. In a tree-grass (living mulch) system, different resource strategies have been widely reported when different growing periods also exist, but their effects have often been ignored. The results of apple and white clover biomass in May, July and October showed that different growing periods of the two crops had a great impact on crop development. The experiment of Zhang et al. proved that with maturation, apple trees will gain advantages in terms of intercropping competition [13]. We proved that this phenomena will not only appear with perennial plants but also with annual crops. In the early stage of white clover development, white clover even was restrained by intercropping competition, which mainly manifested in the decrease of white clover biomass. However, with the development of white clover, white clover gained the dominant position quickly in terms of the competition with apple, inhibiting the development of apple branches, trunks and even leaves. These results confirm that the competitive relationship between intercropped crops will be significantly affected by the growing period of crops or the competitiveness of crops, which could explain why competition in agroforestry systems mainly occurs after the maturity of trees, and the inhibition of a relay cropping system mainly occurs at the beginning of crop development.

According to Figure 2, apple roots under intercropping were different from those of monoculture apple, which could prove a hypothesis (iii): the root morphology of intercropped apples changes significantly due to intercropping. Root morphological characteristics often change with environmental conditions, which may influence carbon allocation and nutrient cycling in the ecosystem [33,34]. Trees under interspecific competition with larger roots systems and more lateral roots have been widely reported in agroforestry systems [35], which strongly supports our hypothesis: we believed intercropped apples will develop roots first at the cost of aboveground development slowing down, to dominant later competition in this type of intercropped system. Higher nutrient accumulation also promotes the further development of the root system, and taking root nitrogen as an example, it is believed that intercropping increases the amount of nitrogen allocated to the root system, results in thinner roots [36,37], consistent with the data on nitrogen. However, there was a strange phenomenon regarding apple roots in the region covered by white clover: in May, there were fewer roots than under monoculture conditions, but in July and October, apple developed many roots in this region. Cover crops can improve soil microbial activity thereby enhancing the nutrient release and affect of the root exudates on the content and accumulated amounts [38,39]. Therefore, we speculate that this was probably because the roots of white clover released some root exudates that activated soil nutrients, which led to the development of apple roots in this region forming a beneficial relationship. The process caused by white clover probably occurred after May, because the development of apple roots in this region was only observed in July.

Orchard grass mulch was a new type of orchard soil management model, and was also a multi-species, multi-level, multi-sequence complex ecosystem constructed artificially, which aims to strengthen the interaction between the components of the composite system. In the present study, the phenomenon that intercropped relationship change the developmental pattern of apple and white clove had been found, but the specific internal mechanism has not been verified. In order to prove this mechanism, biotechnology is an essential tool [22,40,41]. Using biotechnology can reveal the physiological changes of white clover and apple under intercropped relationships at molecular level: it is conceivable that physiological changes must be the basis of changes in developmental patterns.

## Conclusion

In the present study, the apple-white clover intercropped system changed the developmental strategy of the crops which had great significance for the management of orchard cover and the sustainable development of the apple industry. From the changing competitive relationship between the two crops, we found that in arid or semiarid regions, the competitive relationship between two intercropped crops was related to the root development of the crops. Therefore, in the apple-white clover intercropping system, because apple was only 2 years old with an immature root system, white clover inhibited apple development and nutrient accumulation and developed more compared to the responses under monoculture. Under the intercropping systems, decrease intensity of apple biomass was October > July > May, however the increase intensity of white clover biomass was July > October than monoculture systems. However, this growth adversity led apples to focus their development on roots, resulting in apple developed more deeper and wider roots systems, which allowed apples to develop more roots after maturity, thereby increasing the productivity of the entire orchard. In addition, this mismatch in root development is due to the inconsistency in the growing period of the two crops and the intercropping system based on different growing periods would exhibit more advantages under high input levels. Therefore, adequate nutrient supplementation of young fruit trees should be effective in avoiding such competition.

## Highlights

1. Intercropping can result in greater root development and competition for nutrients

2. Seasonal changes can lead to root development and competition for nutrients

3. Adequate nutrient supplementation for the ground cover and crop

4. Rhizo-box experiment
